# Does dietary intake of vitamin A and beta-carotene increase the risk of hypertension?

**DOI:** 10.1097/XCE.0000000000000316

**Published:** 2024-11-14

**Authors:** Sasan Rahmanian, Zahra Salimi, Mohammad Masoumvand, Zohre Aghakhani Nejad, Mohamadtaghi Ghorbani Hesari, Seyed Reza Mirshafaei, Mohammad Keshavarz Mohammadian, Khadijeh Abbasi Mobarakeh, Masoomeh Ataei Kachooei, Ali Shamsi-Goushki, Sara Khoshdooz, Parsa Bahmani, Saeid Doaei, Akram Kooshki, Maryam Gholamalizadeh

**Affiliations:** a Nursing Faculty, Shiraz University of Medical Sciences, Shiraz; b Department of Clinical Nutrition and Dietetics, Student Research Committee, Faculty of Nutrition and Food Technology, Shahid Beheshti University of Medical Sciences, Tehran; c Student Research Committee, Faculty of Medicine, Mashhad University of Medical Sciences, Mashhad; d Department of Nutrition, Faculty of Public Health, Kerman University of Medical Sciences, Kerman; e Mashhad University of Medical Sciences, Mashhad; f Department of Applied Mathematics, Roudsar and Amlash Branch, Islamic Azad University, Roudsar; g Department of Nutrition, Science and Research Branch, Islamic Azad University, Tehran; h Nutrition and Food Security Research Center, Department of Community Nutrition, School of Nutrition and Food Science, Isfahan University of Medical Sciences; i Faculty of Medicine, Shahrekord University of Medical Sciences, Shahrekord; j Department of Nutrition, School of Medicine, Mashhad University of Medical Sciences, Mashhad; k Faculty of Medicine, Guilan University of Medical Sciences, Rasht; l Department of Community Nutrition, National Nutrition and Food Technology Research Institute, Faculty of Nutrition Sciences and Food Technology, Shahid Beheshti University of Medical Sciences, Tehran; m Department of Obstetrics and Gynecology, Reproductive Health Research Center, School of Medicine, Al-Zahra Hospital, Guilan University of Medical Sciences, Rasht; n Non-Communicable Diseases Research Center, Sabzevar University of Medical Sciences, Sabzevar; o Cancer Research Center, Shahid Beheshti University of Medical Sciences, Tehran, Iran

**Keywords:** hypertension, retinol, vitamin A, β-carotene

## Abstract

**Background:**

Hypertension (HTN) is a major global public health issue influenced by genetics and lifestyle factors such as diet and psychological stress. Previous research suggests a potential link between HTN and dietary vitamin A intake. This study aims to explore the association between HTN and the intake of various forms of vitamin A.

**Methods:**

This cross-sectional study was conducted on 1239 patients with HTN and 2945 normotensive individuals aged 35–70 years in Sabzevar, Iran. Dietary vitamin A intake was assessed using the Nutritionist IV software and a food frequency questionnaire.

**Result:**

A positive association was found between HTN with total vitamin A intake [odds ratio (OR): 1.03, 95% confidence interval (CI): 1.01–1.05, *P* = 0.04] and β-carotene intake (OR: 1.03, 95% CI: 1.02–1.05, *P* = 0.03) after adjusting for age and sex. These associations remained statistically significant after adjusting for physical activity and BMI. The association between HTN and β-carotene intake remained significant after additional adjustment for calorie intake. No significant association was observed between dietary retinol intake and HTN.

**Conclusion:**

Increased dietary intake of vitamin A and β-carotene may be associated with a higher risk of HTN. Further longitudinal studies are needed to confirm these findings and elucidate the underlying mechanisms.

## Introduction

Hypertension (HTN) has turned into a major public health concern around the world [1]. Besides prompting noncommunicable diseases, the inability to control HTN may also increase economic and social burdens [2]. HTN is a known risk factor for cardiovascular diseases (CVD), which are responsible for an estimated 7.5 million mortality annually [3]. The worldwide prevalence of HTN among adults is expected to increase up to 1.56 billion cases by 2025 [4]. In the Islamic Republic of Iran, the frequency of HTN in adults over 25 was found to be 25% in women and 24% in males [5].

Genetics, environmental factors, and mental pressure may increase the risk of HTN [4]. HTN autonomously prompts vascular damage and is connected with chronic kidney disease, diabetes, and obesity [6]. Recently, the relationship of nutrients with HTN has been paid extensive attention [7,8]. A healthy diet may have crucial protective roles against HTN [9]. The results of some studies indicated that adherence from some healthy dietary patterns such as low salt diets [10], Mediterranean diet [11], and Dietary Approach to Stop Hypertension diet [12] can be associated with a lower prevalence of high blood pressure (BP). Some important parts of diets that may be connected with HTN are vitamins and phytochemicals [13,14]. For example, vitamin D was frequently assessed as a possible treatment for HTN [15]. It may have this effect via locally triggering macrophages’ renin-angiotensin system, which accelerates atherogenesis. Furthermore, vitamin E is well recognized as a potent antioxidant and anti-inflammatory agent. It has been suggested that vitamin E may have beneficial effects on HTN via different pathways [16].

Carotenoids are generally disseminated in organic products, vegetables, and seaweeds [17], existing in different forms such as precursors of vitamin A (α-carotene and β-carotene), lutein with zeaxanthin, and β-cryptoxanthin [18]. Carotenoids have been shown to exhibit associations with CVD, atherosclerosis, cancer, and several other chronic diseases [19]. Few investigations have been done on the relationship between HTN with carotenoids and vitamin A. Some studies reported the relationship between β-carotene and HTN; however, the results were disputable [20]. A few studies demonstrated that β-carotene can successfully decrease oxidative reactions, consequently diminishing the rate of preeclampsia-related HTN in pregnant women and a decrease of adverse outcomes experienced by neonates and pregnant women [21].

Despite numerous studies, a conclusive link between consuming vitamin A and developing HTN has yet to be established. Numerous research has demonstrated a correlation between increased levels of serum vitamin A and an increased likelihood of developing HTN and heart disease [22,23]. The conflicting findings on the association between HTN and vitamin A might be the result of varying effects from various vitamin A dietary sources. For example, a recent meta-analysis observed an inverse association between total carotenoids and the metabolic syndrome (MetS). However, no association was found between retinol and MetS [24]. So, the aim of this research was to examine the connection between vitamin A and its precursors in the diet and the likelihood of developing HTN.

## Methods

This cross-sectional study was conducted on 4184 participants, including 1239 patients with HTN and 2945 people with normal BP in Sabzevar, Iran. The participants were selected from the prospective epidemiological research studies in Iran (PERSIAN) cohort study, which has been followed from January 2017 to May 2020. The methodological details of the PERSIAN cohort study are published elsewhere [25]. Inclusion criteria included age between 35 and 70 years, no history of kidney diseases, no treatment with drugs affecting BP, no history of vitamin A supplementation, and calorie intake of more than 1000 kcal/day and less than 5500 kcal/day. Exclusion criteria included the inability to collect the required data.

Data on demographic, medical, and nutritional factors were collected by face-to-face interviews and physical examinations. Information related to participants’ economic and social levels was collected by data obtained from the PERSIAN cohort questionnaire. The SECA 204 mobile stadiometer and the SECA 755 mechanical column scale were used to measure height and weight, respectively. To calculate BMI, weight in kilograms was divided by height in meters squared. The level of physical activity was assessed using a validated version of the International Physical Activity Questionnaire [26].

### Dietary intake of vitamin A

Dietary intake data were obtained from the PERSIAN cohort food frequency questionnaire (FFQ), which has already confirmed its validity and reliability [27]. The obtained data on dietary intake were then evaluated using Nutritionist IV software in terms of consumption of different types of vitamin A and β-carotene as the main precursor. In this study, the amount of vitamin A and β-carotene in each food was first determined and then multiplied by the amount of food consumed by each individual.

### Biochemical analysis

Standard mercury sphygmomanometers were used to measure BP based on the mean of three consecutive measures taken after 10 min of rest. Following a night of fasting for the examination, a blood sample was obtained from the participants, and their serum was separated and kept at −70 °C. Data related to BP and blood tests including right systolic BP (SBP), right diastolic BP (DBP), blood urea nitrogen (BUN), triglyceride (TG), alkaline phosphatase (ALP), fasting blood sugar (FBS), serum glutamic oxaloacetic transaminase (SGOT), gamma glutamic transferase (GGT), and serum glutamic pyruvic transaminase (SGPT) were obtained with the coordination of the relevant official.

### Statistical analysis

The Independent *t*-test or its nonparametric counterpart (Mann–Whitney) was utilized for quantitative data, whereas the chi-square test was used for qualitative variables. To investigate the association between high BP and different types of vitamin A in the diet, the logistic regression method and odds ratio estimation were used to analyze the odds of developing the disease using the following models: model 1: adjusted for age and sex; model 2: further adjustments for physical activity, and BMI; and model 3: further adjustments for energy intake. The analyses were conducted using the R studio program with a significance level of *P* < 0.05.

## Results

The mean of age, height, weight, BMI, right SBP, left DBP, FBS, BUN, TG, cholesterol, SGOT, SGPT, ALP, LDL, and GGT, in the normotensive group, were significantly lower than the HTN group. The individuals with normal BP had higher MET and HDL-C than individuals with high BP (all *P* < 0.01) (Table 1).

**Table 1:** Characteristics of the participants

	Normal BP (*N* = 2945)	High BP (*N* = 1239)	*P*
Age (year)	47.46 ± 8.35	53.45 ± 8.3	<0.001
Sex			
Male	1166 (39%)	700 (56%)	<0.01
Female, *N* (%)	1779 (61%)	539 (43%)	
MET final	38.84 ± 7.8	38.1 ± 7.7	0.008
Height (cm)	161.75 ± 9.1	163.32 ± 9.5	<0.001
Weight (kg)	72.51 ± 12.8	78.01 ± 14.2	<0.001
BMI (kg/m^2^)	27.76 ± 4.7	29.23 ± 4.7	<0.001
Right SBP (mmHg)	106.23 ± 10.6	134.12 ± 13	<0.001
Right DBP (mmHg)	67.33 ± 7.8	82.95 ± 7.6	<0.001
FBS (mg/dl)	103.1 ± 35.51	116.89 ± 49.27	<0.001
BUN (mmol/L)	13.46 ± 3.6	14.35 ± 3.9	<0.001
Creatinine (μmol/L)	1.07 ± 0.18	1.14 ± 0.28	<0.001
TG (mg/dl)	137.88 ± 92	166.58 ± 121.3	<0.001
Cholesterol (mg/dl)	189.9 ± 39.06	195.48 ± 42.62	<0.001
SGOT (U/L)	19.89 ± 8.63	20.91 ± 9.59	<0.001
SGPT (U/L)	21.31 ± 15.41	24.01 ± 14.75	<0.001
ALP (U/L)	217.24 ± 66.04	234.13 ± 71.13	<0.001
HDL-C (mg/dl)	52.87 ± 10.66	51.11 ± 10.47	<0.001
LDL (mg/dl)	109.89 ± 33.02	111.23 ± 35.3	<0.001
GGT (U/L)	24.09 ± 21.16	28.85 ± 24.21	<0.001

ALP, alkaline phosphatase; BUN, blood urea nitrogen; FBS, fasting blood sugar; GGT, gamma glutamic transferase; MET, metabolic equivalent of task; Right DBP, right hand diastolic blood pressure; Right SBP, right hand systolic blood pressure; SGOT, serum glutamic oxaloacetic transaminase; SGPT, serum glutamic pyruvic transaminase; TG, triglyceride.

Regarding the intake of nutrients, the HTN group had higher dietary intake of protein (78.14 ± 25.74 vs. 79.98 ± 26.79 g/day, *P* = 0.003), carbohydrate (407.77 ± 135.06 vs. 425.44 ± 144.70 g/day, *P* = 0.001), energy (2477.77 ± 770.36 vs. 2549.93 ± 818.39 kcal/day, *P* = 0.001), total vitamin A (704.57 ± 469.27 vs. 732.99 ± 441.78 mcg/day, *P* = 0.01), and β-carotene (3914.44 ± 2526.62 vs. 4304.08 ± 3080.8, *P* = 0.001) than the normotensive group. Dietary intake of total fat (64.58 ± 25.06 vs. 64.31 ± 25.06 g/day, *P* = 0.01) in the normotensive group was higher than in the HTN group. In terms of retinol, no significant difference was found between the groups (Table 2).

**Table 2:** Dietary intake of the participants

	Normal BP	High BP	*P*
Protein (g/day)	78.14 ± 25.74	79.98 ± 26.79	0.003
Total lipid fat (g/day)	64.58 ± 25.06	64.31 ± 25.06	0.01
Carbohydrate (g/day)	407.77 ± 135.06	425.44 ± 144.70	0.001
Energy (kcal/day)	2477.77 ± 770.36	2549.93 ± 818.39	0.001
Total vitamin A (mcg/day)	704.57 ± 469.27	732.99 ± 441.78	0.01
Retinol (mcg/day)	337.68 ± 360.02	329.18 ± 276.5	0.05
β-carotene (mcg/day)	3914.44 ± 2526.62	4304.08 ± 3080.8	0.001

BP, blood pressure.

Table 3 presents the connections between HTN and the intake of total vitamin A, retinol, and β-carotene. The results revealed a marginal positive association between HTN with a total intake of vitamin A [odds ratio (OR): 1.03, confidence interval (CI): 1.01–1.05, *P* = 0.04] and β-carotene (OR: 1.03, CI: 1.02–1.05, *P* = 0.03) after adjusting for age and sex. These associations remained significant even after adjusting for physical activity and BMI. However, no significant link was observed between HTN and total intake of vitamin A after adjusting for calorie intake. Also, no significant association was found between HTN and dietary intake of retinol.

**Table 3:** The association of hypertension and dietary vitamin A

	Total vitamin A		Retinol		β-carotene	
	OR (95% CI)	*P*	OR (95% CI)	*P*	OR (95% CI)	*P*
Model 1	1.02 (1.01–1.05)	0.04	0.99(0.99–1.01)	0.31	1.02(1.01–1.05)	0.03
Model 2	1.02(1.01–1.05)	0.04	0.99(0.99–1.01)	0.31	1.03(1.01–1.05)	0.03
Model 3	1.01(0.99–1.02)	0.06	0.99(0.98–1.01)	0.33	1.02(1.01–1.05)	0.04

Model 1: adjusted for age and sex; model 2: further adjustments for physical activity and BMI; and model 3: further adjustments for energy intake.

CI, confidence interval; OR, odds ratio.

## Discussion

The present cross-sectional study observed a marginally significant association between higher dietary intakes of total vitamin A and β-carotene with HTN. Furthermore, there was no association between retinol intake and the risk of HTN. The most appropriate and significant precursor for vitamin A is all-trans-β-carotene [28]. This is partly because of its symmetrical structure since all-trans-β-carotene is the only carotenoid that can cleave the central 15,15′ carbon-carbon bond in an oxidative reaction catalyzed by -carotene monooxygenase to produce two molecules of all-trans-retinal. Other geometrical isomers appear less effectively cleaved based on in vitro research [29]. The majority of previous studies revealed a significant association between dietary β-carotene intake and decreased BP [30]. For example, Hozawa *et al*. [31] claimed that there is an inverse relationship between carotenoid concentration with systolic BP and the incidence of HTN. Similarly, Li *et al*. [32] observed that total carotenoid intake of more than 100 mg/kg per day has a beneficial effect on lowering BP in the US population. Besides, another article conducted on 11 336 adults clearly reported that a higher concentration of all six carotenoids may reduce the prevalence of HTN [33]. One of the primary mechanisms supposed in these studies which may contribute to the effect of carotenoids on HTN is the antioxidant properties shown by β-carotene [16]. If the production of reactive oxygen species (ROS) and the body’s antioxidant defenses are not in equilibrium, oxidative stress may result in the onset of HTN. Therefore, one possible factor in the decrease of BP is the ability of β-carotene to eliminate ROS and alleviate oxidative stress [16,34]. Another reason that may be related is that antioxidants cause an increase in nitric oxide production, which has a role in blood vessel dilation and lowers BP [35].

In contrast, the present research indicated that increased β-carotene consumption may be associated with elevated levels of BP. In line with this study, Lv *et al*. [36] stated that women with preeclampsia in their pregnancy had a higher level of vitamin A than normal pregnant women. The contributing mechanism behind it could be an act of β-carotene as a pro-oxidant at high concentrations, which can lead to oxidative stress and inflammation. This, in turn, can damage blood vessels and contribute to the development of HTN [37]. Some investigations confirmed that dependent upon redox potential and other biochemical properties, β-carotene may act as both an antioxidant and a pro-oxidant [38].

Also, no association between dietary intake of retinol with BP was observed. Consistent, another community-based study did not observe any significant association between vitamin A intake and HTN in adults [39]. However, a cohort perspective study demonstrated that a higher intake of vitamin A, either animal-derived or plant-derived, can have a presentational role against HTN [40]. Further studies are required since it is still unclear how precisely retinol, β-carotene, and total intake of vitamin A affect BP. About the result of this study, it is worth mentioning that many different factors, including genetics, lifestyle habits, and other dietary components influence BP. These other factors may mask the effects of retinol and vitamin A on BP. It is also possible that the effects of these nutrients may interact with the effects of vitamin A. For example, a cross-sectional study conducted on the Japanese population suggested that β-carotene may influence the genetic predisposition to developing HTN [41].

It is important to note that this study is just one piece of evidence with some limitations. For example, observational studies are unable to confirm cause-effect relations. In addition, we could not measure or adjust the effect of some confounders, such as the serum level of vitamin A. Besides, the FFQ was applied for evaluating dietary intake, which depends on long-term memory. On the other hand, some of the strength points are included that there was access to large sample size, all measurements were done by trained people and in the standard method, and the effect of different confounders was adjusted in the different models to increase the validity and reliability of the results.

### Conclusion

The current study highlights that dietary intake of vitamin A, retinol, and β-carotene has no beneficial effect against HTN. Furthermore, a higher intake of vitamin A and β-carotene may have an adverse impact on the risk of HTN. To confirm this finding and obtain knowledge of the underlying mechanisms, we need to conduct further longitudinal studies. If the results obtained in the present study are confirmed in future studies, limiting vitamin A intake to the recommended amounts may be considered a BP control strategy in people at risk of HTN.

## Acknowledgements

This study was funded by Sabzevar University of Medical Sciences, Sabzevar, Iran (Code 99219).

The research was approved by the Research Ethics Committee of the Sabzevar University of Medical Sciences, Sabzevar, Iran (Code: IR.MEDSAB.REC.1400.021).

### Conflicts of interest

There are no conflicts of interest.

## References

[1] Mills KT Stefanescu A He J. The global epidemiology of hypertension. Nat Rev Nephrol 2020; 16:223–237. 32024986 10.1038/s41581-019-0244-2 PMC7998524

[2] Upadhyay RK. Chronic non-communicable diseases: risk factors, disease burden, mortalities and control. Acta Scientific Medical Sciences (ISSN: 2582-0931). 2022;6(4.

[3] World Health Organization. A global brief on hypertension: silent killer, global public health crisis: World Health Day 2013. 2013 (WHO/DCO/WHD/2013.2.

[4] Kearney PM Whelton M Reynolds K Muntner P Whelton PK He J. Global burden of hypertension: analysis of worldwide data. Lancet 2005; 365:217–223. 15652604 10.1016/S0140-6736(05)17741-1

[5] Oori MJ Mohammadi F Norozi K Fallahi-Khoshknab M Ebadi A Gheshlagh RG. Prevalence of HTN in Iran: meta-analysis of published studies in 2004-2018. Curr Hypertens Rev 2019; 15:113–122. 30657043 10.2174/1573402115666190118142818 PMC6635676

[6] Berry JD Dyer A Cai X Garside DB Ning H Thomas A. Lifetime risks of cardiovascular disease. N Engl J Med 2012; 366:321–329. 22276822 10.1056/NEJMoa1012848 PMC3336876

[7] Liu M Zhou C Zhang Z Li Q He P Zhang Y. Inverse association between riboflavin intake and new-onset hypertension: a nationwide cohort study in China. Hypertension 2020; 76:1709–1716. 33131313 10.1161/HYPERTENSIONAHA.120.16211

[8] Borghi C Tsioufis K Agabiti-Rosei E Burnier M Cicero AFG Clement D. Nutraceuticals and blood pressure control: a European Society of Hypertension position document. J Hypertens 2020; 38:799–812. 31977574 10.1097/HJH.0000000000002353

[9] Li B Li F Wang L Zhang D. Fruit and vegetables consumption and risk of hypertension: a meta-analysis. J Clin Hypertens (Greenwich) 2016; 18:468–476. 26826021 10.1111/jch.12777 PMC8032179

[10] Cicero AFG Veronesi M Fogacci F. Dietary intervention to improve blood pressure control: beyond salt restriction. High Blood Press Cardiovasc Prev 2021; 28:547–553. 34533781 10.1007/s40292-021-00474-6 PMC8590666

[11] Rahimlou M Grau N Banaie-Jahromi N Taheri M Khosravi A Mavrommatis Y Mohammadifard N. Association of adherence to the dietary approach to stop hypertension and Mediterranean diets with blood pressure in a non-hypertensive population: results from Isfahan Salt Study (ISS). Nutr Metab Cardiovasc Dis 2022; 32:109–116. 34893410 10.1016/j.numecd.2021.09.029

[12] Hashemi R Rahimlou M Baghdadian S Manafi M. Investigating the effect of DASH diet on blood pressure of patients with type 2 diabetes and prehypertension: randomized clinical trial. Diabetes Metab Syndr 2019; 13:1–4. 30641678 10.1016/j.dsx.2018.06.014

[13] Alissa EM Ferns GA. Dietary fruits and vegetables and cardiovascular diseases risk. Crit Rev Food Sci Nutr 2017; 57:1950–1962. 26192884 10.1080/10408398.2015.1040487

[14] Li Z Wang W Liu H Li S Zhang D. The association of serum zinc and copper with hypertension: a meta-analysis. J Trace Elem Med Biol 2019; 53:41–48. 30910205 10.1016/j.jtemb.2019.01.018

[15] Morvaridzadeh M Sepidarkish M Fazelian S Rahimlou M Omidi A Ardehali SH. Effect of calcium and vitamin D co-supplementation on blood pressure: a systematic review and meta-analysis. Clin Ther 2020; 42:e45–e63. 32067744 10.1016/j.clinthera.2020.01.005

[16] Baradaran A Nasri H Rafieian-Kopaei M. Oxidative stress and hypertension: possibility of hypertension therapy with antioxidants. J Res Med Sci 2014; 19:358–367 25097610 PMC4115353

[17] Britton G. Structure and properties of carotenoids in relation to function. FASEB J 1995; 9:1551–1558. 8529834

[18] Chuyen HV Eun JB. Marine carotenoids: bioactivities and potential benefits to human health. Crit Rev Food Sci Nutr 2017; 57:2600–2610. 26565683 10.1080/10408398.2015.1063477

[19] Maiani G Castón MJ Catasta G Toti E Cambrodón IG Bysted A. Carotenoids: actual knowledge on food sources, intakes, stability and bioavailability and their protective role in humans. Mol Nutr Food Res 2009; 53:S194–S218. 19035552 10.1002/mnfr.200800053

[20] Huang G Niu T Peng S Ling D Liu J Zhang X Xu X. Association between the interleukin-1beta C(-511)T polymorphism and blood pressure in a Chinese hypertensive population. Immunol Lett 2004; 91:159–162. 15019285 10.1016/j.imlet.2003.11.009

[21] Talaulikar V Manyonda I. The myth of vitamins C and E for the prevention of preeclampsia: just when will the penny drop? Am J Obstet Gynecol 2010; 203:e7–8; author reply e8. 10.1016/j.ajog.2010.06.063 20723875

[22] Li X Zhu S Song G Zhang K Gao W Huang J Lu X. Retinol-binding protein 4 is closely correlated to blood pressure level and E/A in untreated essential hypertension patients. Ann Palliat Med 2019; 8:645–650. 31865725 10.21037/apm.2019.11.07

[23] Inoue S Takamoto N Akahori Y Masumoto A Nakatsukasa H Msuyama H Hiramatsu Y. Elevated level of serum retinol-binding protein 4 in pregnancy-induced hypertension. J Obstet Gynaecol Res 2009; 35:293–300. 19708176 10.1111/j.1447-0756.2008.00950.x

[24] Beydoun MA Chen X Jha K Beydoun HA Zonderman AB Canas JA. Carotenoids, vitamin A, and their association with the metabolic syndrome: a systematic review and meta-analysis. Nutr Rev 2019; 77:32–45. 30202882 10.1093/nutrit/nuy044 PMC6277204

[25] Poustchi H Eghtesad S Kamangar F Etemadi A Keshtkar A-A Hekmatdoost A. Prospective epidemiological research studies in Iran (the PERSIAN cohort study): rationale, objectives, and design. Am J Epidemiol 2018; 187:647–655. 29145581 10.1093/aje/kwx314 PMC6279089

[26] Moghaddam MB Aghdam FB Jafarabadi MA Allahverdipour H Nikookheslat SD Safarpour S. The Iranian Version of International Physical Activity Questionnaire (IPAQ) in Iran: content and construct validity, factor structure, internal consistency and stability. World Appl Sci J 2012; 18:1073–1080.

[27] Mirmiran P Esfahani FH Mehrabi Y Hedayati M Azizi F. Reliability and relative validity of an FFQ for nutrients in the Tehran lipid and glucose study. Public Health Nutr 2010; 13:654–662. 19807937 10.1017/S1368980009991698

[28] Krinsky NI Johnson EJ. Carotenoid actions and their relation to health and disease. Mol Aspects Med 2005; 26:459–516. 16309738 10.1016/j.mam.2005.10.001

[29] Nagao A Olson JA. Enzymatic formation of 9-cis, 13-cis, and all-trans retinals from isomers of β-carotene. FASEB J 1994; 8:968–973. 8088462 10.1096/fasebj.8.12.8088462

[30] Huang Y Chen H Su Y Liu H Hu J Hong K. Increased blood alpha-carotene, all-trans-beta-carotene and lycopene levels are associated with beneficial changes in heart rate variability: a CVD-stratified analysis in an adult population-based study. Nutr J 2021; 20:1–10. 33971890 10.1186/s12937-021-00700-w PMC8111755

[31] Hozawa A Jacobs DR Jr Steffes MW Gross MD Steffen LM Lee D-H. Circulating carotenoid concentrations and incident hypertension: the Coronary Artery Risk Development in Young Adults (CARDIA) study. J Hypertens 2009; 27:237–242. 19155781 10.1097/HJH.0b013e32832258c9 PMC2920800

[32] Li Z Chen J Zhang D. Association between dietary carotenoid intakes and hypertension in adults: National Health and Nutrition Examination Survey 2007–2014. J Hypertens 2019; 37:2371–2379. 31356404 10.1097/HJH.0000000000002200

[33] Zhu X Shi M Pang H Cheang I Zhu Q Guo Q. Inverse association of serum carotenoid levels with prevalence of hypertension in the general adult population. Front Nutr 2022; 9:971879. 36245540 10.3389/fnut.2022.971879 PMC9563225

[34] do Nascimento TC Cazarin CBB Marostica MR Jr Mercadante AZ Jacob-Lopes E Zepka LQ. Microalgae carotenoids intake: influence on cholesterol levels, lipid peroxidation and antioxidant enzymes. Food Res Int 2020; 128:108770. 31955741 10.1016/j.foodres.2019.108770

[35] Varadharaj S Kelly OJ Khayat RN Kumar PS Ahmed N Zweier JL. Role of dietary antioxidants in the preservation of vascular function and the modulation of health and disease. Front Cardiovasc Med 2017; 4:64. 29164133 10.3389/fcvm.2017.00064 PMC5671956

[36] Lv J Wang Y Zhao Y He Y Yang H Zhang H Wang X. Plasma levels of vitamin A in early pregnancy and correlationship with hypertensive disorder. Comput Math Methods Med 2022; 2022:1–5. 10.1155/2022/3081720 PMC9132624 35633926

[37] Sotler R Poljšak B Dahmane R Jukić T Pavan Jukić D Rotim C. Prooxidant activities of antioxidants and their impact on health. Acta Clin Croat 2019; 58:726–736. 32595258 10.20471/acc.2019.58.04.20 PMC7314298

[38] Palozza P Serini S Di Nicuolo F Piccioni E Calviello G. Prooxidant effects of β-carotene in cultured cells. Mol Aspects Med 2003; 24:353–362. 14585306 10.1016/s0098-2997(03)00031-1

[39] Llopis-González A Rubio-López N Pineda-Alonso M Martín-Escudero JC Javier Chaves F Redondo M. Hypertension and the fat-soluble vitamins A, D and E. Int J Environ Res Public Health 2015; 12:2793–2809. 25749317 10.3390/ijerph120302793 PMC4377933

[40] Zhang Y Liu M Zhou C Zhang Z He P Li Q. Inverse association between dietary vitamin A intake and new-onset hypertension. Clin Nutr 2021; 40:2868–2875. 33940400 10.1016/j.clnu.2021.04.004

[41] Yanagisawa A Suzuki K Kimura A Ito Y Hamajima N Inoue T. Possible protective effect of serum β-carotene levels on the association between interleukin-1B C-31T polymorphism and hypertension in a Japanese population. Clin Nutr 2009; 28:198–202. 19249142 10.1016/j.clnu.2009.01.020

